# Influence of dietary carbohydrate profile on the dairy cow rumen meta-proteome

**DOI:** 10.3168/jds.2022-21812

**Published:** 2022-08-24

**Authors:** B. K. Mulakala, K. M. Smith, M. A. Snider, A. Ayers, M. C. Honan, S. L. Greenwood

**Affiliations:** 1 Department of Animal and Veterinary Sciences, University of Vermont, Burlington 05405; 2 William H. Miner Agricultural Research Institute, Chazy, NY 12921; 3 Department of Animal Science, University of California, Davis 95616

**Keywords:** rumen fermentable starch, physically effective undigested neutral detergent fiber, rumen microbes, proteomics

## Abstract

Diet starch and fiber contents influence the rumen microbial profile and its fermentation products, yet no information exists about the effects of these dietary carbohydrate fractions on the metabolic activity of these microbes. The objective of this experiment was to evaluate the effects of dietary carbohydrate profile changes on the rumen meta-proteome profile. Eight cannulated Holstein cows were assigned to the study as part of a 4 × 4 Latin square design with a 2 × 2 factorial treatment arrangement including four 28-d periods. Cows received 1 of 4 dietary treatments on a dry matter (DM) basis. Diets included different concentrations of rumen fermentable starch (RFS) and physically effective undigested NDF (peuNDF240) content in the diet: (1) low peuNDF240, low RFS (LNLS); (2) high peuNDF240, low RFS (HNLS); (3) low peuNDF240, high RFS (LNHS); and (4) high peuNDF240, high RFS (HNHS). Rumen fluid samples were collected from each cow on the last 2 d of each period at 3 time points (0600, 1000, and 1400 h). The microbial protein fraction was isolated, isobarically labeled, and analyzed using liquid chromatography combined with tandem mass spectrometry techniques. Product ion spectra were searched using the SEQUEST search on Proteome Discoverer 2.4 (Thermo Scientific) against 71 curated microbe-specific databases. Data were analyzed using PROC MIXED procedure in SAS 9.4 (SAS Institute Inc.). A total of 138 proteins were characterized across 26 of the searched microbial species. In total, 46 proteins were affected by treatments across 17 of the searched microbial species. Of these 46 proteins, 28 were affected by RFS content across 13 microbial species, with 20 proteins having higher abundance with higher dietary RFS and 8 proteins having higher abundance with lower dietary RFS. The majority of these proteins have roles in energetics, carbon metabolism, and protein synthesis. Examples include pyruvate, phosphate dikinase (*Ruminococcus albus* SY3), 30S ribosomal protein S11 (*Clostridium aminophilum*), and methyl-coenzyme M reductase subunit α (*Methanobrevibacter ruminantium* strain 35063), which had higher abundances with higher dietary RFS. Conversely, glutamate dehydrogenase (*Butyrivibrio fibrisolvens*) and 50S ribosomal protein L5 (*Pseudobutyrivibrio ruminis*) and L15 (*Ruminococcus bromii*) had lower abundances with higher dietary RFS content. Among the remaining 18 proteins unaffected by RFS content alone, 5 proteins were affected by peuNDF240 content, and 13 were affected by peuNDF240 × RFS interactions. Our results suggest that the RFS content of the diet may have a greater influence on rumen microbial protein abundances than dietary peuNDF240 content or peuNDF240 × RFS interactions. This research highlights that dietary carbohydrate profile changes can influence rumen microbial protein abundances. Further research is needed to fully characterize the effects of diet on the rumen meta-proteome and manipulate the various roles of rumen microbes. This will aid in designing the strategies to maximize the efficiency of nutrient use in the rumen.

## INTRODUCTION

Carbohydrates form the basis of a ruminant’s diet and are necessary for synthesizing body tissues and animal products (McDonald, 2002). As carbohydrates are the primary nutrient in ruminant diets, they directly affect rumen microbial populations. In vitro and in vivo studies support the suggestion that availability of carbohydrates in the rumen is the critical factor controlling the energy availability for rumen microbes (Koenig et al., 2003; Zhang et al., 2015). Availability directly depends on the type of carbohydrate fraction and structure present, which, in turn, influences ruminal fermentation kinetics and dynamics (Villalba et al., 2006). Although starch is a readily fermentable source of energy for rumen microbes and is a crucial component to support the energetic demands of high-yielding dairy cows, dietary NDF and its physical characteristics, such as particle size and digestibility, are the main factors responsible for ruminal health and DMI regulation (Conrad et al., 1964). Physically effective NDF and the NDF fraction undigested after a 240-h in vitro fermentation (**uNDF240**) are essential indicators of rumen function and health. Smith (2019) proposed a method to combine fiber’s physical and chemical nature into a single measurement, termed physically effective uNDF240 (**peuNDF240**). Further, the author reported that the inclusion of peuNDF240 within ration characterization further identifies pertinent fiber fractions and their effects on rumen function. Formulating rations with an optimal balance between NDF fractions and rumen fermentable starch (**RFS**) is critical to optimize digestion, nutrient utilization, and productivity.

Previous studies have investigated the effects of dietary contents of fiber and starch on the rumen microbial profile and activity via meta-genomic and meta-transcriptomic approaches (Thoetkiattikul et al., 2013; Ogunade et al., 2019). The results of these studies reported that feeding a high-fiber diet increased the abundance of *Firmicutes*, whereas feeding a high-starch diet resulted in an increased abundance of *Bacteroidetes* and enriched the genes related to carbohydrate, amino acid, energy, vitamin, and cofactor metabolism pathways. Although these results identify potential impacts of dietary carbohydrates on the rumen microbial profiles, they do not identify the specific fiber or starch fractions being modified. Additionally, many rumen microbes have multiple functions and niches; the results of these studies do not reflect specific microbial activity, as transcript abundance does not necessarily correlate to protein expression.

Identifying and quantifying proteins from rumen microbial communities will lead to meaningful complementary information characterizing the metabolic activity of rumen microbes in response to different diets. This information is critical for further optimization of rumen function and improvement of nutrient use efficiency. This could be achieved using meta-proteomics, an analytical approach that supports the characterization of the entire protein complement within the microbial environment (Hart et al., 2018) and provides a snapshot of microbial activity at a given point in time. Although proteomics technologies are not without limitations, the use of this omics approach can expand characterization and biological understanding. Limitations of the meta-proteomics approach include the following: (1) limited available databases for protein identification, (2) high false discovery rate from the use of large databases, and (3) search engine sensitivity (Ngom et al., 2019; Andersen et al., 2021). However, with advanced sensitivity and accuracy in mass spectrometry analysis and the development of proteomics software platforms, the meta-proteomics approach is a useful tool to develop a deeper insight into the functional and taxonomic interplay of proteins in complex microbial communities and its implications for host health and the environment (Schiebenhoefer et al., 2019). Previous research by Snelling and Wallace (2017) and Hart et al. (2018) provided the first glimpses of microbe-specific proteins in the rumen using gel-based proteomic techniques. Recent modifications by Honan and Greenwood (2020) eliminated the use of gels to expand the breadth of proteins identified in the rumen meta-proteome. This modified approach was applied in this study as a tool to characterize shifts in the meta-proteome in response to dietary carbohydrate profile changes. We hypothesized that dietary carbohydrate profile changes would affect the abundance of proteins associated with energetics and protein synthesis-associated pathways in cellulolytic and amylolytic microbes because of changes in the availability of primary substrates for their growth and production. As little information has been published exploring the effects of dietary carbohydrate profile changes on the rumen microbial meta-proteome profile, this study aims to evaluate the effects of varying concentrations of dietary RFS and peuNDF240 content on rumen microbial protein abundances in Holstein dairy cows using non-gel-based proteomic techniques.

## MATERIALS AND METHODS

### Animals and Experimental Design

Samples analyzed in this experiment were collected from a larger study conducted at the William H. Miner Agricultural Research Institute (Chazy, NY) from July 2019 to September 2019. Experimental procedures were conducted as per protocol approved by the William H. Miner Agricultural Research Institute Animal Care and Use Committee. Eight ruminally cannulated lactating Holstein cows (85 ± 15 DIM) were included in a 4 × 4 Latin square experiment with a 2 × 2 factorial treatment design consisting of four 28-d periods. Cows were paired randomly at the beginning of each period and assigned randomly to 1 of 4 dietary treatments (DM basis) of different concentrations of RFS and peuNDF240 content in the diet. Each cow was exposed to each dietary treatment once. Blinding was not used. Dietary treatments were as follows: (1) low peuNDF240, low RFS (6.35% of DM, 16.7% of DM; **LNLS**); (2) high peuNDF240, low RFS (8.60% of DM, 16.7% of DM; **HNLS**); (3) low peuNDF240, high RFS (6.7% of DM, 19.2% of DM; **LNHS**); and (4) high peuNDF240, high RFS (8.00% of DM, 19.2% of DM; **HNHS**). Dietary CP (as percent of DM) of the LNLS, HNLS, LNHS, and HNHS diets were 16.1%, 16.0%, 15.3%, and 15.2%, respectively. The complete list of diet ingredients, chemical composition, and particle size distribution, as outlined by Smith (2021), can be found in Supplemental Tables S1 and S2 (https://data.mendeley.com/datasets/wymtjsncg2/1; Mulakala, 2022). Briefly, different concentrations of peuNDF240 were achieved through inclusion of either brown midrib-3 or conventional corn silages, and the different concentrations of RFS were achieved with different inclusion amounts of corn meal. Diet peuNDF240 was calculated by multiplying the physically effective factor by the uNDF240 organic matter content of the diet that was retained on a ≥1.18-mm sieve. The RFS of the diet was calculated by multiplying the starch digestibility measured at 7 h by the starch content of the diet. Smith (2021) reported that total-tract digestibility of NDF was higher with the low-peuNDF240 diets compared with feeding a high-peuNDF240 diet. Smith (2021) also observed a lack of effects of dietary treatments of peuNDF240 or RFS, or their interaction, on DMI, rumen fermentation profiles, or rumen pH.

### Rumen Fluid Sampling

Rumen fluid samples for this experiment were collected from each cow on d 27 (1400 h) and d 28 (0600 h and 1000 h) of each period. Two samples from one cow during the second period (0600 h and 1000 h) and 8 samples from period 4 (1000 h) were not available for analysis. At each of the 3 sampling time points, rumen fluid samples were collected from 3 locations in the mid-ventral ruminal sac of each cow and composited within time point within the cow, as previously described (Steele et al., 2012). Samples (n = 1 sample per cow per time point) were flash-frozen immediately after each collection using a dry ice ethanol bath, transported on dry ice to the laboratory, and stored at −80°C until processing.

### Protein Extraction from Rumen Fluid

Frozen rumen fluid samples (n = 96) were thawed overnight at 4°C. Once thawed, samples were strained through 4 layers of cheesecloth (Lion Services Inc.) to separate coarse fibers. Samples were then pooled within cow within the period across the 3 points, creating 1 composite sample per cow per period (n = 32 samples). A universal control (**UC**) was generated by pooling equal volumes of rumen fluid from each cow from each time point across the 4 periods. The samples and UC were then centrifuged at 16,000 × *g* for 20 min at 4°C to collect the pellet.

The collected pellets were processed as Honan and Greenwood (2020) described. Briefly, radioimmunoprecipitation assay (RIPA) lysis buffer containing protease inhibitor (Pierce Protease Inhibitor Mini Tablets, Thermo Scientific) and a 5-mm stainless steel bead (Qiagen) were added to collected pellets. Samples were homogenized (TissueLyser II, Qiagen) at 30 Hz for 30 s followed by a 3-min incubation on ice, and this homogenization/incubation process was repeated for a total of 6 repetitions. The resulting homogenate samples were then precipitated by adding a 6 *M* trichloroacetic acid/80 m*M* dithiothreitol solution at a 1:3 protein extract ratio. The samples were then vortexed and incubated overnight at 4°C. Following the overnight incubation, samples were vortexed and centrifuged at 16,000 × *g* for 20 min at 4°C. Pellets were washed twice with ice-cold 20% dimethyl sulfoxide in acetone and twice with 100% ice-cold acetone. Precipitated pellets were air-dried and resuspended in PBS. The total protein extract concentrations were quantified using a bicinchoninic acid assay kit (Pierce).

### Tandem Mass Tag Peptide Labeling

Tandem mass tag (**TMT**) peptide labeling (an isobaric label) was performed as per manufacturer instructions (Thermo Scientific). Briefly, 72 μg of protein from each sample was aliquoted, and the volume of each sample was normalized to 100 μL with 100 m*M* triethylammonium bicarbonate. Samples were then reduced with 0.5 m*M* dithiothreitol for 60 min at 37°C, followed by iodoacetamide alkylation in the dark for 30 min. Trypsin was then added at a 1:50 (wt/wt) ratio, and the samples were digested overnight at 37°C. Dissolved TMT reagents were added to the digested samples and incubated for 1 h, subsequently quenched with 8 μL of 5% hydroxylamine. Multiplexes were created by combining equal volumes (100 μL) of a random subset of individual TMT-labeled samples (a maximum of 9 experimental samples per multiplex) + 1 aliquot of UC per plex. A 100-μL aliquot from each plex (total 5 plexes) was dried to remove triethylammonium bicarbonate. Peptides were then fractionated using a high-pH reversed-phase peptide fractionation kit (Thermo Scientific) as per kit instructions, resulting in 8 fractions for each plex. Fractionated plex samples were submitted to the Vermont Biomedical Research Network Proteomics Facility (University of Vermont, Burlington, VT) for liquid chromatography-tandem MS analysis.

### Liquid Chromatography-Tandem MS Analysis

Protein identification and quantification were performed on a Q-Exactive Plus MS coupled to an EASYnLC 1200 (Thermo Scientific), similar to the workflow described by Honan and Greenwood (2020). Briefly, the fractionated peptide samples were dissolved in 10 μL of 2.5% formic acid (**FA**) and 2.5% acetonitrile (**ACH**) in water and then loaded onto a 100-μm × 500-mm capillary column packed with Halo C18 (2.7-μm particle size, 90-nm pore size, Michrom Bioresources) at a flow rate of 300 nL/min. The column end was laser pulled to a ~3-μm orifice and packed with a minimal amount of 5-μm MAGIC C18AQ before packing with the 3-μm particle size chromatographic materials. Solvent gradient conditions were as follows: 2.5 to 35% of ACH/0.1% FA for 60 min; increase from 35 to 100% ACH/0.1% FA in 1 min and then 100% ACH/0.1% FA for 4 min; immediate return to 2.5% ACH/0.1% FA; and a final hold at 2.5% ACH/0.1% FA. Peptides were introduced into the MS via a nanospray ionization source with a spray voltage of 2.0 kV. Full MS scans were acquired in a data-dependent “Top 10” acquisition mode with lock mass function activated (*m*/*z* 371.1012). The scans were acquired in the mass range of 300 to 1,600 *m*/*z* with a mass resolution of 70,000, and the automatic gain control target value was set at 1e^6^. The top 10 intense peaks in MS were fragmented with higher-energy collisional dissociation of 10. The liquid chromatography-tandem MS spectra were obtained at a 35,000 resolution, with an automatic gain control target of 1e^5^ and a maximum injection time of 100 ms. Dynamic exclusion was enabled (peptide match: preferred; exclude isotopes: on; underfill ratio: 1%).

### Data Analysis

Product ion spectra were searched using the SEQUEST search engine with percolator node (false discovery rate set to less than 1%) on Proteome Discoverer 2.4 (Thermo Scientific) against 71 composite databases downloaded on September 12, 2020, from UniProt. Methionine oxidation (+15.995 Da) and TMT6plex modification on N-termini and lysine (+229.163 Da) were chosen as variable modifications, and cysteine alkylation by iodoacetamide was selected as a fixed modification. Precursor mass ion tolerance was defined at ±10 ppm and fragment mass ion tolerance at ±0.02 Da. The protease was specified as trypsin, with 2 maximum missing cleavages. The TMT-labeled peptides were quantified with the Reporter Ions Quantifier node in the consensus workflow, and parameters were specified as follows: unique, and razor peptides were used for quantification; Reject Quan results with missing channels and apply Quan value corrections were set as false; Co-Isolation Threshold value was set as 50; Average Reporter S/N Threshold was 10; “Total Peptide Amount” was used for normalization, and Scaling Mode was set “on Control Averages,” so that the peptide abundances in the UC labels were set as 100 and the abundances in other channels were scaled accordingly. The scaled abundance values of the samples were used for further statistical analyses. For protein identification, 71 microbial composite proteome databases were manually selected for peptide comparison searches in SEQUEST. Microbes to be included in this analysis were selected because of their known prevalence in the rumen and their involvement in rumen microbial carbohydrate metabolism. The SEQUEST engine then comparatively searched protein and peptide masses detected in the sample against the selected 71 composite proteome databases by statistical matching to identify proteins, their microorganism, and strain source. For any proteins identified as uncharacterized, the FASTA sequence was retrieved from UniProt (http://www.uniprot.org/) and searched through the Basic Local Alignment Search Tool (BLAST; http://blast.ncbi.nlm.nih.gov/Blast.cgi). The top hit protein was selected as an identified protein if the hit was 100% matched with the searched query. A mixed procedure model of SAS 9.4 (SAS Institute Inc.) was performed, including peuNDF240, RFS, peuNDF240 × RFS interactions, replicate, and cow(replicate) as the fixed effects and cow as the random effect. The effect of diet on protein abundance was considered significant if *P* < 0.05.

## RESULTS

In this study, the meta-proteomic analysis identified 138 proteins labeled in all samples across 26 of the searched microbial species (Supplemental Table S3; https://data.mendeley.com/datasets/wymtjsncg2/1). The following microbes and strains were identified: *Prevotella ruminicola*, *Prevotella ruminicola strain* ATCC 19189, *Prevotella bryantii*, *Ruminococcus albus* 8, *Ruminococcus albus* strain 27210, *Ruminococcus albus* strain SY3, *Ruminococcus bromii*, *Butyrivibrio hungatei*, *Prevotella aff. ruminicola* Tc2–24, *Methanobrevibacter ruminantium* strain 35063, *Eubacterium cellulosolvens* 6, *Ruminococcus flavefaciens*, *Pseudobutyrivibrio ruminis*, *Selenomonas ruminantium*, *Eubacterium ruminantium*, *Fibrobacter succinogenes strain* ATCC 19169, *Treponema bryantii*, *Pseudobacteroides cellulosolvens* ATCC 35603, *Clostridium aminophilum*, *Oxalobacter formigenes* HOxBLS, *Megasphaera elsdenii* DSM 20460, *Treponema saccharophilum*, *Butyrivibrio fibrisolvens*, *Butyrivibrio proteoclasticus* strain ATCC 51982, *Selenomonas ruminantium* ssp. *lactilytica* strain NBRC 103574, and *Methanosarcina barkeri*.

The diet treatments affected 46 proteins across 17 of the searched microbial species. Of these 46 proteins, 28 proteins were affected by RFS content, 5 were affected by peuNDF240 content, and 13 were affected by peuNDF240 × RFS interactions.

### Microbial Protein Profile Affected by RFS Content in the Diet

Dietary RFS content affected 28 microbial proteins (63% of affected proteins; Table 1), with 20 proteins having higher abundance with higher dietary RFS and 8 proteins having higher abundance with lower dietary RFS. These proteins were associated with 13 searched microbial species: *P. ruminicola*, *P. ruminicola strain* ATCC 19189, *P. aff. ruminicola* Tc2–24, *R. albus* SY3, *M. ruminantium* strain 35063, *B. fibrisolvens*, *B. hungatei*, *R. albus* 8, *C. aminophilum*, 6, *Pseudobacteroides cellulosolvens* ATCC 35603, *Pseudobutyrivibrio ruminis*, *E. cellulosolvens* and *R. bromii.*

### Microbial Protein Profile Affected by peuNDF240 Content in the Diet

Of the 138 proteins identified in this experiment, only 5 microbial proteins were affected by peuNDF240 content (Table 2), with 4 proteins having higher abundance with higher dietary peuNDF240 and 1 protein having higher abundance with lower dietary peuNDF240. These proteins originated from 3 microbial species: pyruvate, phosphate dikinase, and sn-glycerol-3-phosphate ABC transporter ATP-binding protein UgpC from *R. bromii*; 30S ribosomal protein S5 from *Ruminococcus* sp.; and acyl-CoA dehydrogenase from *E. cellulosolvens* 6 had higher abundances with higher dietary peuNDF240 content. Conversely, 30S ribosomal protein S16 from *P. ruminicola* had a lower abundance with higher dietary peuNDF240 content.

### Microbial Protein Profile Affected by peuNDF240 × RFS Interactions in the Diet

Thirteen microbial proteins were affected by peuNDF240 × RFS interactions (Table 3). These proteins were associated with 10 searched microbial species: *C. aminophilum*, *R. bromii*, *P. ruminicola*, *T. bryantii*, *P. bryantii*, *Pseudobutyrivibrio ruminis*, *S. ruminantium*, *B. fibrisolvens*, *Prevotella* sp., and *P. ruminicola* strain ATCC 19189.

## DISCUSSION

In the present study, not surprisingly, higher numbers of proteins were identified from microbial genera that are more completely cataloged in the available databases. Additionally, many proteins were identified across multiple species, likely resulting from conserved sequences or similar functional roles. The 5 proteins most highly identified across microbial species were elongation factor Tu, 30S and 50S ribosomal proteins, GAPDH, and glutamate dehydrogenase (**GDH**). These results were not surprising, given that elongation factor Tu accounts for 5 to 10% of total cell protein (Weijland et al., 1992), and ribosomal proteins account for 20% of bacterial dry weight (Russell, 2002). In line with these observations, Hart et al. (2018) reported elongation factor Tu, GAPDH, and GDH as all being in the top 25 most abundant proteins found within the bovine rumen. Similarly, Honan and Greenwood (2020) reported that elongation Tu and ribosomal proteins were widely represented proteins across the microbial species characterized in their study. Given the importance of GAPDH in glycolysis and the essential role of GDH in enabling ammonia assimilation within the rumen and microbial protein synthesis (Wallace et al., 1997), the high prevalence of these proteins is also not surprising.

### Effects of Diet on the Rumen Meta-Proteome

**Figure 1.**
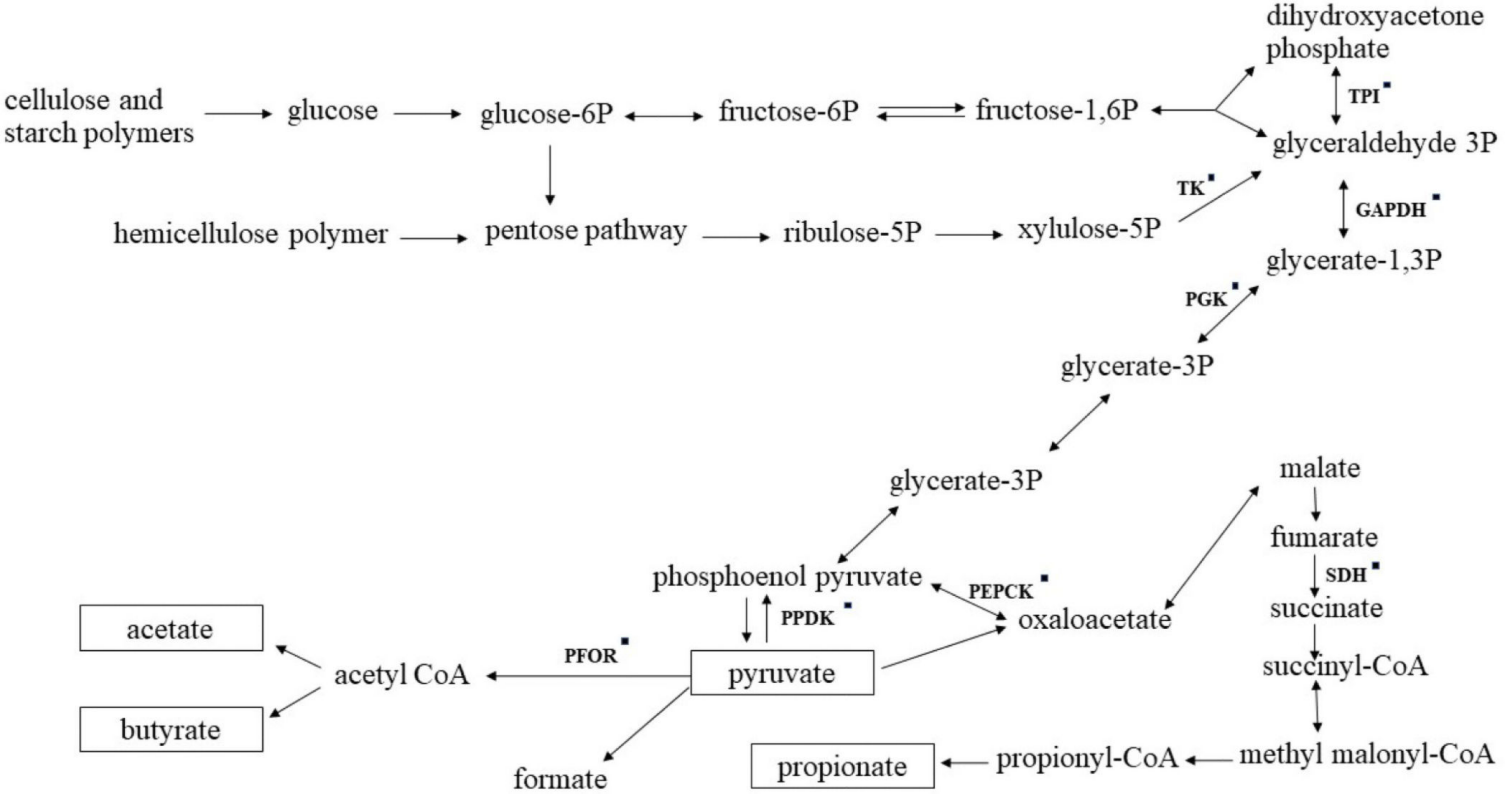
Metabolic pathways involved in the degradation of carbohydrates in the rumen (adapted from Russell, 2002; Wirth et al., 2018). Proteins identified in this study are indicated by black squares (■). Unidirectional arrows indicate that the protein catalyzes a reaction in one direction, whereas bidirectional arrows indicate that the protein can catalyze forward and reverse reactions. TPI = triosephosphate isomerase; GAPDH = glyceraldehyde 3-phosphate dehydrogenase; TK = transketolase; PGK = phosphoglycerate kinase; PFOR = pyruvate ferredoxin oxidoreductase; PPDK = pyruvate phosphate dikinase; PEPCK = phosphenol pyruvate carboxy kinase; SDH = succinate dehydrogenase.

Altered dietary carbohydrate profile affected the abundance of 46 microbial proteins in the present study, representing 33% of identified proteins. Not surprisingly, most of these proteins were functionally associated with energetics (Figure 1), protein biosynthesis, and carbon metabolism pathways, but, interestingly, many of these proteins were primarily associated with the genus *Prevotella*. At the ingredient level, a previous rumen meta-proteomic study reported that abundances of proteins assigned to the *Prevotellaceae* family were not affected by the provision of different feedstuffs, including comparisons of corn silage versus grass silage versus grass hay fed to Jersey cows (Deusch et al., 2017). However, studies have reported a shift in the abundance of *Prevotella* in response to diet nutrient profiles. For example, Zhang et al. (2014) reported that primary dietary forage sources affected the abundance *Prevotella.* In their study, intake of an alfalfa diet increased the proportion of the *Prevotella* genera compared with the proportion observed in cows fed a cornstalk diet. Additionally, Thoetkiattikul et al. (2013) and Zeng et al. (2019) reported an increased abundance of *Prevotella* in cows fed a high-starch diet. These differences might be due to changes in nutrient profiles, as *Prevotella* can utilize a range of carbohydrates, from simple mono- or oligosaccharides to complex plant polysaccharides such as pectins, mannans, starch, and hemicelluloses (Miyazaki et al., 1997; Matsui et al., 2000).

### Influence of RFS Content on the Rumen Meta-Proteome

Rumen fermentable starch content had a much greater effect on the characterized rumen meta-proteome compared with peuNDF240. In the current study, 28 proteins, representing 63% of affected proteins, were affected due to RFS content. Many of these proteins were associated with (1) energetics, (2) carbon metabolism, and (3) protein biosynthesis pathways.

Glyceraldehyde-3-phosphate dehydrogenase is involved in oxidative phosphorylation of D-glyceraldehyde-3-phosphate to 1,3-bisphosphate-D-glycerate (Ferri et al., 1978), a central step in glycolysis. *Butyrivibrio hungatei* GAPDH abundance was higher with lower dietary RFS content, which is not unexpected, given that *B. hungatei* is a secondary degrader species that utilizes fiber degradation as substrates for growth (Kopečný et al., 2003). *Prevotella ruminicola* GAPDH abundance was higher with lower dietary RFS content. In contrast, *P. ruminicola* pyruvate:ferredoxin (flavodoxin) oxidoreductase (**PFOR**) was higher with higher dietary RFS content. Oxidative decarboxylation of pyruvate to form acetyl-coenzyme A is catalyzed by PFOR, a crucial step in many metabolic pathways. The shift in the abundance of *P. ruminicola*’s GAPDH and PFOR may be part of *P. ruminicola*’s versatile behavior in response to different dietary substrates available in the rumen. As *Prevotella* constitutes one of the most predominant genera in the rumen, with major roles in carbohydrate metabolism, further research is needed to explore the regulation of carbohydrate metabolism associated protein in *Prevotella* in response to different dietary substrates’ availability and concentrations.

The abundances of phosphoglycerate kinase, pyruvate phosphate dikinase (**PPDK**), and triosephosphate isomerase from *R. albus* were higher in response to higher dietary RFS content. Phosphoglycerate kinase and anaerobic PPDK are involved in glycolysis and gluconeogenesis pathways. Phosphoglycerate kinase catalyzes the reversible interconversion of 1,3-bisphosphoglycerate and 3-bisphosphoglycerate. Anaerobic PPDK catalyzes the reversible reactions from phosphoenolpyruvate to pyruvate (Chastain et al., 2011). The increase in abundance of these proteins from *R. albus* with high dietary RFS content may indicate an increase in the rate of gluconeogenesis, as *R. albus* utilizes cellulose as the primary substrate for growth in the rumen, although this was not validated in the current trial. To support this suggestion, we observed the abundance of triosephosphate isomerase, a gluconeogenic protein responsible for catalyzing the interconversion of dihydroxyacetone phosphate to D-glyceraldehyde 3-phosphate, was higher with higher dietary RFS. Transketolase, an enzyme involved in the pentose phosphate pathway of carbohydrate metabolism, is an alternative branch of glycolysis used to produce the sugars that form DNA and RNA (Appel and Parrot, 1970). An increase in the abundance of transketolase with higher dietary RFS may indicate that *P. aff. ruminicola* utilizes starch as the primary substrate for growth. The higher abundance of phosphoglycerate kinase from *P. aff. ruminicola* with higher dietary RFS content further supports the species growth with high RFS content. However, these postulated effects of RFS on *P. aff. ruminicola* require further investigation for validation.

We also observed a higher abundance of *P. ruminicola* succinate dehydrogenase with higher RFS content. Succinate dehydrogenase catalyzes the synthesis of fumarate from succinate, which is required for both aerobic and anaerobic growth. The higher abundance of this protein from *P. ruminicola* with a higher RFS fraction in the diet is in agreement with the previous observation that *Bacteroides* use the succinate pathway for growth (Macy and Probst, 1979). Glutamate dehydrogenase from *B. fibrisolvens* was lower with higher dietary RFS content, whereas GDH from *P. aff. ruminicola* was lower with lower dietary RFS content. Given that GDH is thought to be a major ammonia assimilatory enzyme in the rumen, our results agree with the premise that GDH activity is repressed when rumen bacteria are grown under glucose-limiting conditions (Pengpeng and Tan, 2013). *Butyrivibrio fibrisolvens* utilizes fiber as the primary substrate for growth, whereas *P. aff. ruminicola* uses starch as the primary substrate for growth.

In this study, the higher abundances of the 30S ribosomal proteins S1, S5, and S3, and the 50S ribosomal protein L22 from *P. ruminicola* in response to higher dietary RFS suggest increased *P. ruminicola* protein synthesis. Increased abundance of the 50S ribosomal protein L1 from *R. bromii* with lower dietary RFS content may indicate an increase in microbial biomass with a lower RFS fraction diet, although shifts in biomass were not measured in our current study. It may be because of increased NDF digestibility due to low RFS content in the diet, as *R. bromii* relies on dietary NDF as a substrate to support growth. To support the possible explanation of increased NDF digestibility due to low RFS content in the diet, Smith (2021) reported that higher-RFS diets had a tendency to result in lower NDF digestibility. Further, Ferraretto et al. (2013) observed a decrease in rumen NDF degradability with an increase in dietary starch content. *Pseudobutyrivibrio ruminis* is a fibrolytic bacterium, and Zened et al. (2013) reported a decrease in the abundance of *Pseudobutyrivibrio ruminis* with high-starch diets. An increase in the abundance of 50S ribosomal protein L5 from *Pseudobutyrivibrio ruminis* with lower RFS was not unexpected, but an increase in the abundance of glycine-tRNA ligase with higher RFS content was not expected. The glycine-tRNA ligase catalyzes an amino acid’s attachment to its cognate tRNA molecules and is consequently vital for protein biosynthesis (Kaiser et al., 2020). Previously, Pidcock et al. (2021) reported that *Pseudobutyrivibrio* species contain an abundance of carbohydrate-active enzyme isoforms that facilitate metabolic plasticity and resilience under dietary perturbations. Thus, the observed increase of glycine-tRNA ligase abundance with higher dietary RFS content may result from their metabolic plasticity and resilience characteristics under dietary perturbations.

An increase in the abundance of 30S ribosomal protein S11 from *C. aminophilum* with higher dietary RFS content was observed. Because *C. aminophilum* is an obligatory amino acid fermenting bacterium in the rumen, this could indicate that protein synthesis increases with high RFS fraction diets. Although increased abundance could be due to increased concentration of microbial metabolites in the rumen environment, particularly amino acids and peptides, because of high starch digestibility, this causal relationship is speculative and requires validation. The enzyme D-3-phosphoglycerate dehydrogenase catalyzes the first step in the l-serine biosynthetic pathway by converting D-3-phosphoglycerate, an intermediate metabolite in glycolysis, to phosphohydroxypyruvate (Grant, 2018). A higher D-3-phosphoglycerate dehydrogenase protein abundance from *P. aff. ruminicola* in response to higher dietary RFS content may indicate that protein synthesis is likely increasing with a high diet RFS inclusion. Increased abundance of *P. aff. ruminicola* DNA-directed RNA polymerase subunit α with higher dietary RFS content further supports our suggestion that total protein synthesis by *P. aff. ruminicola* may be increasing in response to a higher dietary RFS content. Higher abundance of *E. cellulosolvens 6* polyribonucleotide nucleotidyl transferase with higher dietary RFS content may indicate that protein synthesis is likely decreasing, as polyribonucleotide nucleotidyl transferase is involved in mRNA degradation. This was not surprising, as *E. cellulosolvens 6* is a fibrolytic bacterium. An increased abundance of polyribonucleotide nucleotidyl transferase may have occurred because *E. cellulosolvens 6* prioritized their functions to pathways requiring lower energy rather than to replication, due to limited substrate availability with a diet higher in RFS content. Also supportive of the suggestion of shifts in protein synthesis was our observation of a higher abundance of *Pseudobacteroides cellulosolvens* heat shock chaperone protein DnaK with lower dietary RFS content, as molecular chaperones exist inside cells as families of proteins with diverse molecular masses (e.g., 90, 70, 60, and 30 kDa) that involve folding and refolding proteins to prevent denaturation.

In the present study, higher abundances of methanogenesis-associated proteins [5,10-methylenetetrahydromethanopterin reductase and methyl-coenzyme M reductase subunit α-component of methyl-coenzyme M reductase (**McrI**)] from *M. ruminantium* were observed with higher dietary RFS content. Although methane measurements were not collected in the current trial, known relationships could provide some insight into this observation. Specifically, the abundance of *M. ruminantium* is typically related to low methane production (Danielsson et al., 2012, 2017). Methyl-CoM reductase exists in 2 isomeric forms, McrI and McrII, with McrI expressed at low hydrogen concentrations and McrII expressed at high hydrogen concentrations (Reeve et al., 1997). With past research identifying that *M. ruminantium* M1 does not code for McrII (Leahy et al., 2010), Danielsson et al. (2017) speculated that *M. ruminantium* might be unable to scavenge hydrogen for methanogenesis in an environment with higher hydrogen concentrations, which may also explain our finding. The increased abundance of McrI and 5,10-methylenetetrahydromethanopterin reductase from *M. ruminantium* likely occurred because increased starch content favors propionate production, thus reducing hydrogen production and methanogenic substrate availability (Hassanat et al., 2013; Rooke et al., 2014; Pirondini et al., 2015). This study further supports the suggestion that the activity of *M. ruminantium* is associated with low methane production.

### Influence of peuNDF240 on the Rumen Meta-Proteome

In the present study, we observed a shift in the abundance of PPDK from *R. bromii* in response to peuNDF240 content in the diet, indicating a relationship between *R. bromii* and dietary fiber shifts. In line with our observation, *R. bromii* has historically been considered fibrolytic in its activity (Deng et al., 2007). An increased abundance of 30S ribosomal protein S16 from *Ruminococcus* spp. in cows fed higher dietary peuNDF240 content suggests that protein synthesis could be increasing; however, this was not directly measured or validated in the current study. This may indicate that *Ruminococcus* spp. growth is likely increasing with higher dietary peuNDF240 content, due to increase in the availability of NDF for fermentation, likely resulting from an increased rumen NDF retention rate.

A higher abundance of acyl-CoA dehydrogenase from *E. ruminantium* was also observed with higher dietary peuNDF240 content. This enzyme catalyzes the first step of fatty acid β-oxidation, and the rate of fatty acid oxidation is high during fasting but low in the fed animal (Schulz, 2013). *Eubacterium ruminantium* is a primary cellulolytic bacterium; therefore, an increase in abundance of acyl-CoA dehydrogenase from *E. ruminantium* with higher dietary peuNDF240 content may be a result of shifting NDF degradability, as high uNDF240 diets have less NDF degradability, which may have ultimately limited the substrate availability for *E. ruminantium* fermentation. As only 5 proteins were affected by peuNDF240, it appears that (1) peuNDF240 contents were too similar across diets in the current study to create a microbial activity response; (2) response variables other than protein abundance—for example, enzymatic rate—may be the more appropriate variables to assess when studying the effects of uNDF240 on microbial metabolism; or (3) other microbes not included in this characterization may need to be considered in future studies.

### Influence of peuNDF240 × RFS Interactions on the Rumen Meta-Proteome

Thirteen microbial proteins were affected by the interaction of peuNDF240 × RFS; all are associated with carbohydrate metabolism and protein biosynthesis pathways. Some interactive effects are not unexpected, as studies have shown that physically effective NDF interacts with starch degradation kinetics, modifying the responses of ruminal fermentation and performance in dairy cows. For example, Zebeli et al. (2008) reported that the passage rate of rumen degradable starch was accelerated from the reticulorumen when the physically effective NDF content of the diet was increased. Additionally, Yang and Beauchemin (2006) have shown that feeding longer-size fiber particles shifted starch digestion from the rumen into the intestine, lowering the development of acidosis.

The function of phosphoenolpyruvate carboxykinase (**PEPCK**) is to catalyze oxaloacetate conversion to phosphoenolpyruvate as part of the gluconeogenesis pathway. A higher abundance of PEPCK from *S. ruminantium* in cows fed with the LNLS diet compared with those fed the HNHS diet was observed. This may be because of decreased substrate availability and, ultimately, energy availability associated with the LNLS diet, as *S. ruminantium* is an amylolytic bacteria. Supporting this observation, Asanuma and Hino (2001) reported that the mRNA expression of PEPCK was inversely related to the sufficiency of energy in *S. ruminantium*, suggesting that PEPCK synthesis is regulated at the transcriptional level when energy supply was altered. A higher abundance of *P. bryantii* PFOR with HNHS and LNLS compared with the HNLS diet may indicate the species’ growth with HNHS and LNLS diets. A higher abundance of acyl-CoA dehydrogenase from *B. fibrisolvens* in response to the HNHS diet compared with the HNLS and LNLS diets may be due to decreased primary substrate availability for energy production by *B. fibrisolven*s, as this is primarily a cellulolytic bacterium. Further research is required to confirm this postulated relationship.

The abundance of 30S ribosomal protein S3 from *R. bromii* was higher in response to the HNLS diet compared with the LNLS or HNHS diets. Additionally, the abundance of elongation factor from *R. bromii* was higher with HNLS compared with the LNLS diet. These observations may indicate that protein synthesis in *R. bromii* increases with the HNLS diet. Although any shift in protein synthesis requires further validation, in support of this suggestion we also observed an increased abundance of 50S ribosomal proteins L1 from *R. bromii* with the HNLS diet. The abundance of proteins related to protein biosynthesis, such as pseudouridine synthetase, 60 kDa chaperonin, and 30S ribosomal protein S12 from *P. ruminicola* was affected by peuNDF240 × RFS. However, the abundances were varied, with no clear trend across treatments. This is somewhat expected, as *Prevotella* has a remarkable degree of genetic diversity (Purushe et al., 2010) and therefore can occupy various ecological niches within the rumen (Jami and Mizrahi, 2012). These observations also further support the characteristic metabolic versatility of *P. ruminicola*, enabling them to adapt to different dietary changes in the rumen.

## CONCLUSIONS

In this experiment, we identified 138 rumen microbial proteins and the shifts in their abundance relative to peuNDF240 and the RFS content of the diet. Alteration of dietary carbohydrate profiles affected 46 proteins across 17 of the searched microbial species, mainly due to RFS content. Many of the proteins affected by diet have known functions related to energetics, protein biosynthesis, and carbon metabolism. This research demonstrates the breadth and specificity of shifts in protein-mediated microbial activity in the rumen with altered carbohydrate substrate availability. Additionally, our results highlight the need to further examine and characterize the rumen meta-proteome in relation to the diet, to improve our understanding of rumen microbial responsiveness. Furthermore, this is among the first research contributing to the identification of diet-directed mechanisms that affect core microbial metabolic pathways and ruminant productivity using proteomic approaches.

## Figures and Tables

**Table 1.** Rumen microbial protein abundances of Holstein dairy cows affected by high and low dietary concentrations of rumen fermentable starch

Accession no.	Protein	Species	HRFS^1^	LRFS^2^	*P*-value

A0A011V1T4	Pyruvate, phosphate dikinase	*Ruminococcus albus* SY3	98	86	0.018
A0A0L6JLZ7	Chaperone protein DnaK	*Pseudobacteroides cellulosolvens* ATCC 35603	63	97	0.001
A0A1G5DIH3	Glyceraldehyde-3-phosphate dehydrogenase	*Butyrivibrio hungatei*	77	95	0.001
A0A1G5IE59	50S ribosomal protein L15	*Ruminococcus bromii*	74	99	<0.001
A0A1H5ULG9	Glyceraldehyde-3-phosphate dehydrogenase	*Prevotella ruminicola*	113	127	0.043
A0A1H5VSL2	Pyruvate ferredoxin/flavodoxin oxidoreductase	*Prevotella ruminicola*	96	76	0.020
A0A1H5WM35	Elongation factor G	*Prevotella ruminicola*	108	122	0.011
A0A1H5WWH7	30S ribosomal protein S3	*Prevotella ruminicola*	98	81	0.025
A0A1H7FGA7	Glycine-tRNA ligase	*Pseudobutyrivibrio ruminis*	110	93	0.006
A0A1I0LYZ3	Transketolase	*Prevotella aff. ruminicola*	147	96	0.005
A0A1I0M5Y7	D-3-Phosphoglycerate dehydrogenase	*Prevotella aff. ruminicola* Tc2–24	102	94	0.023
A0A1I0MM67	Phosphoglycerate kinase	*Prevotella aff. ruminicola* Tc2–24	86	65	0.032
A0A1I0P5C6	Glutamate dehydrogenase	*Prevotella aff. ruminicola*	103	95	0.024
A0A1I0P5I5	DNA-directed RNA polymerase subunit α	*Prevotella aff. ruminicola* Tc2–24	96	75	0.037
A0A1I6IX72	30S ribosomal protein S11	*[Clostridium] aminophilum*	139	107	0.001
A0A1M6U8F7	50S ribosomal protein L22	*Prevotella ruminicola*	93	84	0.014
A0A1M6U920	30S ribosomal protein S5	*Prevotella ruminicola*	110	99	0.024
A0A2G3E9W1	50S ribosomal protein L5	*Pseudobutyrivibrio ruminis*	41	110	0.007
A0A317G314	Glutamate dehydrogenase	*Butyrivibrio fibrisolvens*	93	103	0.016
D3E050	Methyl-coenzyme M reductase subunit α	*Methanobrevibacter ruminantium*	106	85	0.001
D3E1K9	5,10-Methylenetetrahydromethanopterin reductase	*Methanobrevibacter ruminantium*	102	85	0.001
D5EUX2	50S ribosomal protein L11	*Prevotella ruminicola* (strain ATCC 19189)	122	131	0.045
D5EWF4	Succinate dehydrogenase/fumarate reductase, flavoprotein subunit	*Prevotella ruminicola* strain ATCC 19189	117	94	0.007
D5EX27	l-Arabinose isomerase	*Prevotella ruminicola* (strain ATCC 19189)	114	87	0.002
E9SA28	Triosephosphate isomerase	*Ruminococcus albus* 8	116	88	0.034
E9SBQ5	Phosphoglycerate kinase	*Ruminococcus albus* 8	122	86	0.006
I5AW29	Polyribonucleotide nucleotidyltransferase	*[Eubacterium] cellulosolvens*	102	90	0.024
UPI0008EEAC0D	30S ribosomal protein S1	*Prevotella ruminicola*	106	93	0.015

1 HRFS = high rumen fermentable starch (19.2% of DM).

2 LRFS = low rumen fermentable starch (16.7% of DM).

**Table 2.** Rumen microbial protein abundances of Holstein dairy cows affected by low and high dietary concentrations of physically effective undigested NDF after 240 h of in vitro fermentation (peuNDF240)

Accession no.	Protein	Species	High peuNDF240^1^	Low peuNDF240^2^	*P*-value

A0A1H5UBN2	30S ribosomal protein S16	*Prevotella ruminicola*	94	105.4	0.036
A0A1T4JZ88	Acyl-CoA dehydrogenase	*Eubacterium ruminantium*	135	108.0	0.013
A0A412UNB6	Pyruvate, phosphate dikinase	*Ruminococcus bromii*	102	91.3	0.005
A0A414DYV0	Sn-glycerol-3-phosphate ABC transporter ATP-binding protein UgpC	*Ruminococcus bromii*	94	74.3	0.019
UPI00031FF12C	30S ribosomal protein S5	*Ruminococcus*	119	79.7	0.014

1 High peuNDF240 = high physically effective undigested NDF after 240 h of in vitro fermentation (mean ± SE, 8.3 ± 0.30% of DM).

2 Low peuNDF240 = low physically effective undigested NDF after 240 h of in vitro fermentation (6.21 ± 0.14% of DM).

**Table 3.** Rumen microbial protein profile of Holstein dairy cows affected by different dietary concentration levels of physically effective undigested NDF after 240 h of in vitro fermentation (peuNDF240) and rumen fermentable starch

			High peuNDF240^1^		Low peuNDF240^2^		
Accession no.	Protein	Species	HRFS^3^	LRFS^4^	HRFS^3^	LRFS^4^	*P*-value

A0A1G5IDN6	30S ribosomal protein S3	*Ruminococcus bromii*	71^b^	107^a^	92^ab^	79^b^	<0.05
A0A1H5RLC4	Pseudouridine synthase	*Prevotella ruminicola*	85^b^	104^ab^	100^b^	123^a^	<0.05
A0A1H6GDE8	Phosphoenolpyruvate carboxykinase	*Selenomonas ruminantium*	74^b^	81^b^	80^b^	104^a^	<0.05
A0A1H9IAR9	Flagellin	*Treponema bryantii*	117^a^	85^b^	90^ab^	87^b^	<0.05
A0A1I0C755	ABC transporter substrate-binding protein	*[Clostridium] aminophilum*	101^a^	91^ab^	82^b^	101^a^	<0.05
A0A1M7JI43	30S ribosomal protein S12	*Ruminococcus flavefaciens*	70^b^	104^ab^	122^a^	80^ab^	<0.05
A0A2G3EDW8	GGGtGRT protein	*Pseudobutyrivibrio ruminis*	114^ab^	124^a^	99^b^	113^ab^	<0.05
A0A2N0UM52	50S ribosomal protein L1	*Ruminococcus bromii*	115^b^	173^a^	95^b^	109^b^	<0.05
A0A317G571	Acyl-CoA dehydrogenase	*Butyrivibrio fibrisolvens*	188^a^	144^b^	158^ab^	117^c^	<0.05
A0A412LBR6	Elongation factor	*Ruminococcus bromii*	124^ab^	137^a^	129^ab^	109^b^	<0.05
D5EWS5	60 kDa chaperonin	*Prevotella ruminicola* (strain ATCC 19189)	84^b^	80^b^	110^a^	83^b^	<0.05
UPI0001D0765C	30S ribosomal protein S12	*Prevotella*	73^b^	95^ab^	87^b^	101^a^	<0.05
UPI0001DB225B	Ferredoxin oxidoreductase	*Prevotella bryantii*	106^b^	80^b^	101^ab^	110^a^	<0.05

a Mean values in the same row with different superscripts differ (*P* < 0.05).

b Mean values in the same row with different superscripts differ (*P* < 0.05).

1 High peuNDF240 = high physically effective undigested NDF after 240 h of in vitro fermentation (mean ± SE, 8.30 ± 0.30% of DM).

2 Low peuNDF240 = low physically effective undigested NDF after 240 h of in vitro fermentation (6.21 ± 0.14% of DM).

3 HRFS = high rumen fermentable starch (19.2% of DM).

4 LRFS = low rumen fermentable starch (16.7% of DM).
